# Clinical outcomes and associated factors in pediatric patients undergoing tracheostomy in a public intensive care unit

**DOI:** 10.62675/2965-2774.20260345

**Published:** 2026-05-08

**Authors:** Iago Silva de Almeida, Lucieny Silva Martins Serra, Catarina Ferreira Costa Praia, Alexandre Peixoto Serafim

**Affiliations:** 1 Hospital Anchieta Pediatric Intensive Care Unit Brasília DF Brazil Pediatric Intensive Care Unit, Hospital Anchieta - Brasília (DF), Brazil.; 2 Hospital Materno Infantil de Brasília Pediatric Intensive Care Unit Brasília DF Brazil Pediatric Intensive Care Unit, Hospital Materno Infantil de Brasília - Brasília (DF), Brazil.; 3 Universidade de Brasilia Brasília DF Brazil Universidade de Brasilia - Brasília (DF), Brazil.

**Keywords:** Tracheostomy, Tracheitis, Airway obstruction, Child, Palliative care, Hospital, public, Hospital mortality

## Abstract

**Objective:**

To describe clinical outcomes and identify predictors of death and complications in children undergoing tracheostomy at a public referral hospital in the Federal District.

**Methods:**

A retrospective cohort study including 123 children (zero to 14 years) who underwent tracheostomy between 2017 and 2021. Patients under exclusive palliative care, those lost to follow-up, or with a tracheostomy performed prior to 2017 were excluded. Follow-up spanned from the procedure date until decannulation, death, or the study end (August 2, 2025), with a mean duration of 26.9 months. Data on demographics, clinical indicators, complications, and outcomes were analyzed using descriptive statistics, survival analysis (Kaplan-Meier), binary logistic regression, and Poisson regression.

**Results:**

The majority were infants (58.5%) and male (53.7%). Upper airway obstruction was the predominant indication (41.5%). The overall mortality was 39%, and septic shock was the leading cause (16.7%). Complications occurred in 52% of cases, most notably tracheitis (42.2%) and accidental decannulation (32.8%). Multivariate analysis identified the following as independent predictors of death: tracheitis (OR 4.79; 95%CI 2.15 - 10.68; p = 0.001), mechanical ventilation dependence (OR 3.43; 95%CI 1.52 - 7.74; p = 0.003), and accidental decannulation (OR 2.44; 95%CI 1.05 - 5.66; p = 0.037). Poisson regression showed that longer tracheostomy use time (IRR 1.137; p = 0.008) and complex chronic diseases (IRR 1.573; p = 0.011) were associated with higher complication rates.

**Conclusion:**

Tracheostomized children in this public hospital setting experience high morbidity and mortality, influenced significantly by modifiable factors such as infection, ventilator dependence, and decannulation events.

## INTRODUCTION

Pediatric tracheostomy is a crucial procedure in the management of children with chronic respiratory failure or airway obstruction. Recent studies demonstrate that long-term outcomes are determined by a complex interaction between patient-specific factors, underlying pathological processes, and perioperative management.^(1–3)^ The most robust associated factors with mortality and decannulation include the indication for tracheostomy, the presence and severity of comorbidities (particularly neurological impairment and heart disease), ventilator dependence, nutritional status, and age at the time of the procedure.^(4–6)^

Children with severe neurological disability, ventilator dependence at hospital discharge, and congenital heart disease have significantly higher mortality and lower decannulation rates.^(7–9)^ In high-income countries, socioeconomic factors do not appear to significantly impact long-term survival in cohorts with access to intensive care, suggesting that clinical factors predominate in determining outcomes.^(10)^ However, it is hypothesized that in middle- and low-income countries, systemic limitations in healthcare access, outpatient support, and resource allocation may profoundly alter this dynamic, potentially exacerbating the risk associated with both clinical and socioeconomic factors.^(11)^ Therefore, conducting localized studies with real-world data is critical to identify these context-specific disparities and to understand the true determinants of outcomes in resource-constrained settings like Brazil.

To address this gap, we conducted a study with the objective of describing clinical outcomes and identifying predictors of death and complications in children undergoing tracheostomy at a public referral hospital in the Federal District. The findings are intended to generate hypotheses for future research and inform the ongoing discussion on optimizing care for this vulnerable population in a low-resource setting.

## METHODS

### Study design and population

A retrospective cohort study was conducted, including 123 children (zero - 14 years) who underwent a new tracheostomy procedure (incident cases) at the *Hospital Materno Infantil de Brasília* between January 2017 and December 2021. Patients under exclusive palliative care, those lost to follow-up, or those whose tracheostomy was performed prior to 2017 (prevalent cases) were excluded.

### Variables and data collection

Data were retrospectively collected from electronic medical records from the date of tracheostomy until the end of the study follow-up (August 2, 2025). The collected variables included:

– Demographic and clinical characteristics: age, sex, primary indication for tracheostomy.
– Exposure and follow-up variables: duration of tracheostomy use (calculated from the procedure date until decannulation, death, or end of study), mechanical ventilation (MV) dependence (continued need for invasive mechanical ventilatory support beyond the immediate post-operative period, as documented in the medical records at the time of discharge from the intensive care unit or at the last follow-up), neuropsychomotor development, feeding method, outpatient follow-up, and home care.
– Outcome variables: complications (tracheitis, bleeding, accidental decannulation) and mortality. Due to the retrospective nature of this study, the diagnosis of "tracheitis" was based on the attending medical team's clinical documentation in the patient's electronic medical record.

### Statistical analysis

Data was analyzed using STATA 16®. Categorical variables were described as frequencies and percentages, and continuous variables as medians and interquartile ranges.

For inferential analyses, group comparisons used the Mann-Whitney U test, Kruskal-Wallis test, Pearson's Chi-squared test, or Fisher's exact test, as appropriate.

Survival analysis was performed using Kaplan–Meier curves with the log-rank test.

To identify independent predictors of death, a binary logistic regression model was fitted. To ensure model stability and avoid overfitting, we adhered to the "ten events per variable" rule by including only the three most clinically and statistically significant predictors. The final model included death (yes/no) as the dependent variable and the following covariates: MV dependence (yes/no), occurrence of tracheitis (yes/no), and accidental decannulation (yes/no). Results were expressed as adjusted odds ratios (OR) with their respective 95% confidence intervals (95%CI). Model diagnostics were performed, including the Hosmer-Lemeshow goodness-of-fit test, assessment of multicollinearity via variance inflation factor (VIF), and evaluation of discriminatory power via the area under the curve (AUC).

Additionally, to model the complication rate per patient (count data), a Poisson regression model was employed. Independent variables included were: age at tracheostomy (months), duration of tracheostomy use (years), and type of indication (categorical). Results were expressed as incidence rate ratios (IRR) with 95%CI.

A significance level of 5% (α = 0.05) was adopted for all analyses, and confidence intervals were calculated at 95% (95%CI).

### Ethical considerations

Approved by the Research Ethics Committee of the *Fundação de Ensino e Pesquisa em Ciências da Saúde* (FEPCS), under CAAE: 86959424.9.0000.5553 and protocol number: 7.589.951. For families that could be located based on medical records, we obtained the Free and Informed Consent Form by telephone or electronically.

## RESULTS

### Population characteristics

During the 5-year study period (January 2017 to December 2021), there were 2,207 admissions to the pediatric intensive care unit (ICU). From this population, 123 children (zero to 14 years) who underwent a new tracheostomy procedure (incident cases) were identified and comprised our study cohort. No tracheostomized patients meeting the inclusion criteria were excluded from the analysis, resulting in a final analyzed sample of n = 123, the majority of whom were infants (58.5%) and male (53.7%). The predominant indication was upper airway obstruction (41.5%), followed by pulmonary disease (22.8%) and neurological disease (17.9%) (Table 1).

**Table 1:** Demographic and clinical characteristics of children undergoing tracheostomy in a public hospital in the Federal District (2017 - 2021)

Variable		n (%)
Sex		
	Male	66 (53.7)
	Female	57 (46.3)
Age at tracheostomy (years)		
	Infants (< 2)	72 (58.5)
	Preschoolers (2 - 5)	26 (21.1)
	School-aged (> 5)	25 (20.3)
Duration of tracheostomy use (years)		
	< 1	21 (17.1)
	1 - 3	29 (23.6)
	4 - 5	28 (22.8)
	6 - 8	45 (36.6)
Primary indication for tracheostomy		
	Upper airway obstruction	51 (41.5)
	Chronic pulmonary disease	28 (22.8)
	Neurological disease	22 (17.9)
	Complex chronic conditions	12 (9.8)
	Congenital heart disease	10 (8.1)
Outcome		
	Death	48 (39.0)
	Maintains tracheostomy	59 (48.0)
	Successfully decannulated	16 (13.0)

Data expressed as n (%).

### Main outcomes

The overall mortality was 39% (n=48), with septic shock being the main cause (16.7%). Complications occurred in 52% of cases, most notably tracheitis (42.2%), bleeding (35.9%), and accidental decannulation (32.8%). Only 21.1% of patients were fed orally, and 33.3% were dependent on MV.

**Figure 1:**
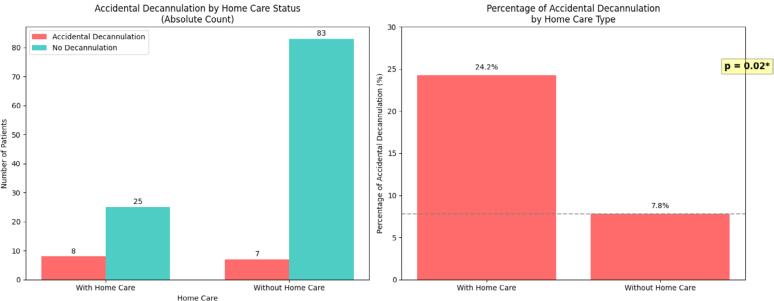
Home care *versus* accidental decannulation.

A statistically significant association was found between receiving home care and a higher occurrence of accidental decannulation (Fisher's Exact Test, p = 0.02) (Figure 1).

### Survival analysis

**Figure 2:**
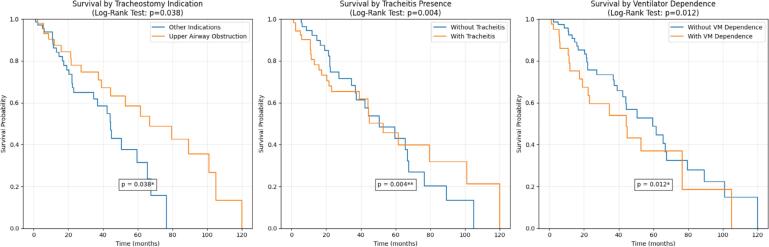
Survival curves according to tracheostomy indication.

The cohort was followed from the date of tracheostomy until decannulation, death, or the censoring date of August 2, 2025. The mean follow-up time for the 123 patients was 26.9 months (standard deviation: ±21.9 months), with a range from 0.2 to 95.8 months. The median survival time was 42 months. Kaplan-Meier analysis demonstrated significant differences in survival according to tracheostomy indication (p = 0.038), presence of tracheitis (p = 0.004), and MV dependence (p = 0.012) (Figure 2).

### Multivariate analysis

Logistic regression identified the following as independent predictors of death: tracheitis (OR 4,55; 95%CI 2,10 - 9,85; p = 0.001), MV dependence (OR 3.25; 95%CI 1,48 - 7,12; p = 0.003), and accidental decannulation (OR 2.52; 95%CI 1.12 - 5.68; p = 0.026) (Table 2). Poisson regression showed an association between longer duration of tracheostomy use (IRR 1.137; p = 0.008) and complex chronic diseases (IRR 1.573; p = 0.011) with a higher number of complications (Table 3). Each additional year of tracheostomy use is associated with a 13.7% increase in the complication rate. Pulmonary disease and complex chronic diseases are associated with 37.0% and 57.3% higher complication rates, respectively, compared to upper airway obstruction.

**Table 2:** Logistic regression: coefficients and significance

Variable	Adjusted odds ratio	95% confidence interval	p value*
Mechanical ventilation dependence	3.25	2.10 - 9.85	0.001
Tracheitis	4.55	1.48 - 7.12	0.003
Accidental decannulation	2.52	1.12 - 5.68	0.026

Model diagnostics: events per variable: 16; Hosmer-Lemeshow Goodness-of-Fit: χ^2^ = 6.12, p = 0.42; discriminatory power (AUC): 0.81 (95%CI: 0.73 - 0.89); multicollinearity (mean variance inflation factor): 1.3.

* NS = not significant (p > 0.05).

**Table 3:** Poisson regression: coefficients and significance

Variable	Coefficient	IRR	95% confidence interval	p value*
Intercept	0.892	2.44	1.42 - 4.18	0.001
Age at tracheostomy (months)	-0.005	0.995	0.987 - 1.004	0.213
Duration of tracheostomy use (years)	0.128	1.137	1.034 - 1.250	0.008
Indication: pulmonary disease	0.315	1.370	1.012 - 1.854	0.042
Indication: neurological disease	0.284	1.328	0.979 - 1.802	0.067
Indication: congenital heart disease	0.192	1.212	0.914 - 1.608	0.185
Indication: complex chronic diseases	0.453	1.573	1.111 - 2.226	0.011

Reference: Upper airway obstruction.

* NS = not significant (p > 0.05).

## DISCUSSION

This study demonstrates high rates of morbidity and mortality in children with tracheostomies at a public hospital in the Federal District. These findings are consistent with the international literature, albeit of a greater magnitude.^(12–14)^ The overall mortality of 39% exceeds the rates reported in high-income countries (20 - 30%),^(15–17)^ reflecting likely specific contextual challenges within the Brazilian Unified Health System (SUS - *Sistema Único de Saúde*). Our results identified modifiable clinical factors, such as tracheitis, MV dependence, and accidental decannulation, as independent predictors of death. At the same time, the duration of tracheostomy use and the presence of complex chronic diseases were associated with a higher incidence of complications.

Tracheitis emerged as the most significant independent risk factor for death (OR = 4.79). This finding underscores the critical importance of rigorous infection prevention and management protocols in the care of these patients. Previous studies, such as Schweiger et al., have also reported tracheitis as a frequent complication in pediatric tracheostomy cohorts.^(12)^ Bacterial colonization and recurrent infections are known determining factors for hospital readmissions, representing a significant burden on the healthcare system and families.^(6,11)^ On the other hand, Morrison et al. state that there is still no standardized definition for tracheostomy-associated respiratory infections, which hinders comparisons between studies and the implementation of universal protocols.^(18)^ The authors emphasize that most studies use criteria based on clinical judgment or hospital coding – a reality similar to that of the present study – and they reinforce the urgent need for prospective trials to guide the diagnosis and treatment of these infections in tracheostomized children. 

Dependence on MV tripled the risk of death, a result that consistently corroborates the international literature.^(1)^ Similarly, the Poisson regression analysis showed that complex chronic diseases were associated with a 57.3% higher rate of complications. These findings reinforce that the burden of underlying comorbidities is a fundamental determinant of long-term prognosis. Other studies, such as those by Berry et al. and McPherson et al., have identified that conditions such as neurological impairment and chronic lung diseases are strongly associated with lower decannulation rates, higher mortality, and greater use of hospital resources.^(2,4)^ The survival analysis in the present study confirmed a worse prognosis in these subgroups. Specifically, within the Brazilian context, we postulate that systemic limitations - such as delays in accessing specialized multiprofessional care, shortages of home ventilatory support equipment, and weaknesses in outpatient follow-up - may exacerbate the risks associated with these complex clinical conditions.

Accidental decannulation doubled the risk of death in the multivariate analysis. This finding likely reflects critical deficiencies in caregiver training and post-discharge support. A statistically significant association was observed between accidental decannulation and the presence of home care. While this association does not imply causality, it raises the hypothesis that cases referred for home care may be of greater complexity, or that there are failures in training these caregivers to manage emergencies. The literature reports accidental decannulation rates of 8% to 20%.^(19,20)^ Strategies such as caregiver-focused realistic simulation, the use of safety sutures, and the provision of home emergency kits are cited in the literature as potential mitigating measures.^(19–21)^ National studies, such as Pereira et al. in Acre, highlight the insecurity experienced by caregivers in low-resource contexts, who often have to improvise with supplies or rely on emergency services for issues that could be managed at home with adequate training.^(22)^ However, it should be noted that it was done in a distinct socio-economic context. Our data therefore support the need for more studies on structured training programs and continuous support for families, tailored to local socioeconomic realities.

The Poisson regression analysis indicated that each additional year of tracheostomy use was associated with a 13.7% increase in the complication rate. This temporal relationship was expected, as prolonged exposure to the tube increases the risk of events such as granulomas, stenoses, and infections. Veder et al. also observed that a longer tracheostomy duration was significantly associated with a higher risk of persistent tracheocutaneous fistula after decannulation.^(10)^ This finding reinforces the importance of regular endoscopic reviews and the systematic and early attempt at decannulation whenever clinically indicated, to minimize the time with the cannula and, consequently, the cumulative risk of complications.

### Limitations and direction of bias

The limitations of this study include its retrospective design and single-center sample. Furthermore, the loss of follow-up of some participants and incomplete medical records may have influenced the results. It is plausible that patients with more severe outcomes or from families with greater social vulnerability were more prone to loss the follow-up, which could underestimate the true rates of mortality and complications. Alternatively, if more complex cases had more frequent follow-up and more detailed records, the inverse effect could occur. The observational nature of the data prevents causal inference, and the implications discussed should be viewed as hypothesis-generating for future research, preferably prospective and multicenter, to better control for these biases and investigate the causal relationships suggested here.

## CONCLUSION

Children with tracheostomies in a public hospital in the Federal District present high morbidity and mortality, with modifiable clinical factors – tracheitis, mechanical ventilation dependence, and accidental decannulation – significantly influencing outcomes. The observed associations between worse outcomes and contexts of greater vulnerability (e.g., home care) suggest that targeted strategies for infection prevention, robust and continuous caregiver training, strengthening home care, and structured outpatient follow-up are critical areas for intervention.

## Data Availability

After publication the data will be available on demand to authors.
